# Cervical Neuroendocrine Carcinoma: A Rare Case Report

**DOI:** 10.7759/cureus.15532

**Published:** 2021-06-08

**Authors:** Tarun Kumar, Jitendra S Nigam, Madhu Kumari, Swati ., Jagjit Pandey

**Affiliations:** 1 Pathology/Lab Medicine, All India Institute of Medical Sciences, Patna, IND; 2 Surgical Oncology, All India Institute of Medical Sciences, Patna, IND

**Keywords:** cervix, neuroendocrine, pap smear, carcinoma, small cell

## Abstract

Neuroendocrine carcinoma is a rare tumor in the uterine cervix with a dismal prognosis. Clinically, it is difficult to differentiate from other cervical malignancies. Clinical presentation varies from vaginal bleeding, discharge per vaginum and cervical mass. For better clinical outcomes, it is vital to diagnose promptly and accurately. We report a 35-year-old female presented with whitish discharge per vaginum and lower abdominal pain for six months. Per speculum reveals an irregular, firm mass measuring 4x3 cm involving both the cervical lips, which turned out to a small cell neuroendocrine carcinoma.

## Introduction

Cervix cancer is the second most common cancer constituting 22.86% of all cancer cases in Indian women [1]. Neuroendocrine (NE) neoplasms (NENs) are NE cell-derived malignancies that can occur in various parts of the body, including the female genital tract [2]. NENs of the cervix are uncommon, divided into NE tumors (NETs) and NE carcinomas (NECs) [3]. Both NETs and NECs have a similar clinical presentation, such as vaginal bleeding, discharge, and cervical mass, as seen in other cervical malignancies but have variable prognoses [4]. NETs constitute low-grade epithelial neoplasm (Grade 1 and Grade 2) [3]. Small cell NE carcinoma (SCNEC) and large cell NE carcinoma (LCNEC) are included in high-grade carcinoma [3]. Therefore, early and correct diagnosis is of utmost importance because of its aggressive behavior, dismal prognosis, and specific treatment modalities [5]. Herein we are presenting this case due to its rarity in a young female having a diagnostic pitfall.

## Case presentation

**Figure 1:**
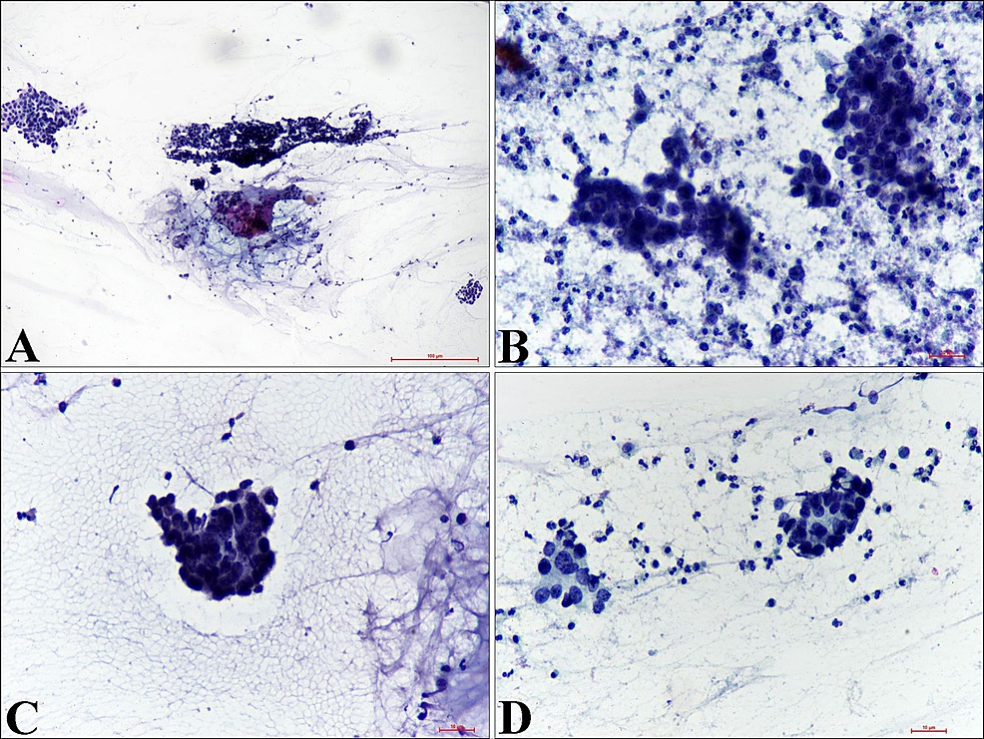
Cervical Pap Smear Cytology Examination (A) Clusters of atypical cells (Papanicolaou stain, x100). (B) Malignant cells with high nuclear-cytoplasmic ratio and hyperchromatic nuclei in a necrotic background (Papanicolaou stain, x400). (C) Malignant cells displaying nuclear molding (Papanicolaou stain, x400). (D) Occasional acinar arrangement (Papanicolaou stain, x400).

A 35-year-old female presented to the gynecology outpatient department with a complaint of whitish discharge per vaginum and lower abdominal pain for six months. The menstrual history was unremarkable. Per speculum reveal an irregular, firm mass measuring 4x3 cm involving both the lips of the cervix. Clinical suspicion of carcinoma cervix, International Federation of Gynaecology and Obstetrics (FIGO) stage 2A was made. Pap smear reveals a cellular smear showing atypical cells arranged in a small cluster and few singly scattered, having increased nuclear-cytoplasmic ratio, scant cytoplasm, and coarse to hyperchromatic nuclei. Pseudo-rosette-like glandular differentiation and occasional nuclear molding are also noted (Figures 1A-1D).

**Figure 2:**
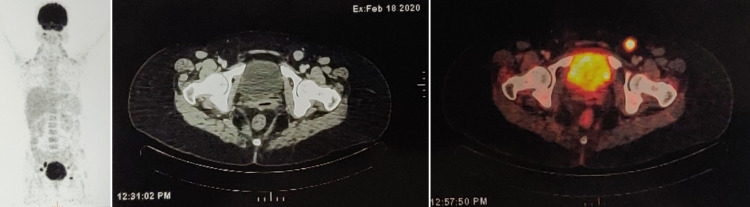
Whole-Body Fluorodeoxyglucose F-18 Positron Emission Tomography Scan Active hypermetabolic soft tissue thickening in the cervix with left inguinal, pelvic, and mesenteric lymph node metastasis.

The background shows necrosis and acute inflammatory cells. A cytological diagnosis of atypical - glandular cells, favor neoplastic was suggested. Whole-body F-18 labeled fluorodeoxyglucose (FDG)-positron emission tomography (PET) scan reveals an active hypermetabolic soft tissue thickening in the cervix. The left inguinal, pelvic, and mesenteric lymph nodes showed metastatic evidence (Figure 2).

**Figure 3:**
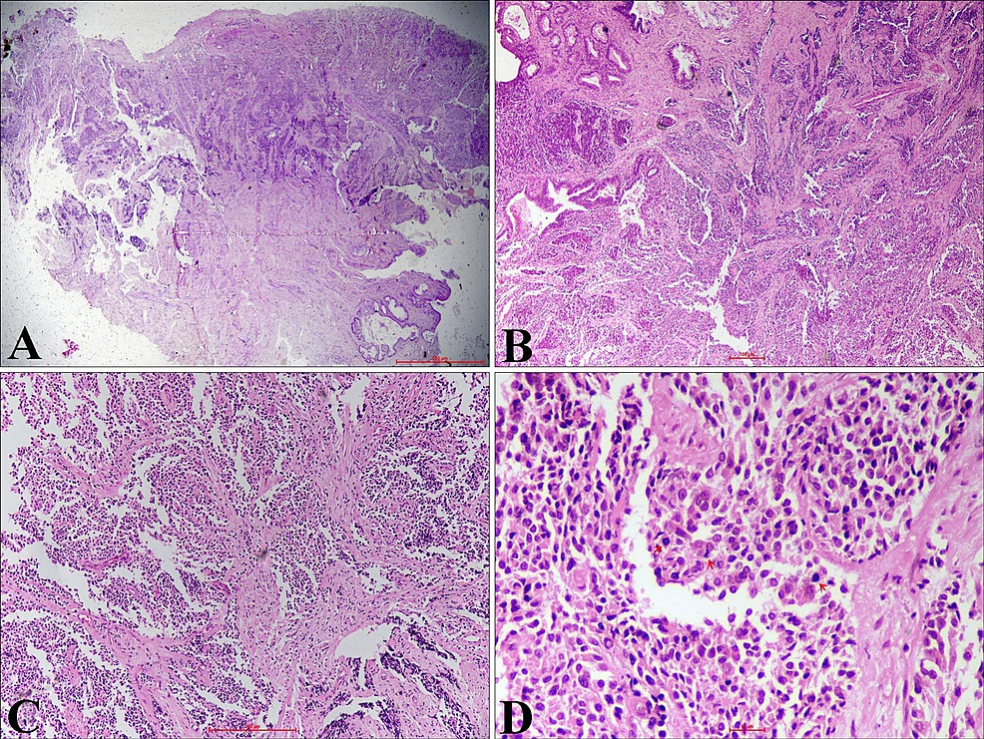
Hematoxylin and Eosin-Stained Sections From Cervical Tissue (A) Tumor with cervical epithelial ulceration (H&E, x20). (B, C) Stroma infiltrated by tumor cells arranged in diffuse sheets and vague nested pattern (H&E, x40, x100). (D) Monomorphic, small to round tumor cells having hyperchromatic nuclei, high nuclear-cytoplasmic ratio, and scant cytoplasm. Atypical mitosis is also noted (arrow) (H&E, x400). H&E - Hematoxylin and eosin

Biopsy from the lesion shows tumor cells arranged in the nest, lobule, and diffuse pattern. The tumor is composed of monomorphic, round to oval cells. These cells were having a high nuclear-cytoplasmic ratio, hyperchromatic nuclei, inconspicuous nucleoli, and scant cytoplasm. Few cells showed stippled chromatin and nuclear molding. Occasional atypical mitosis and focal crushing artifact are also noted (Figures 3A-3D).

**Figure 4:**
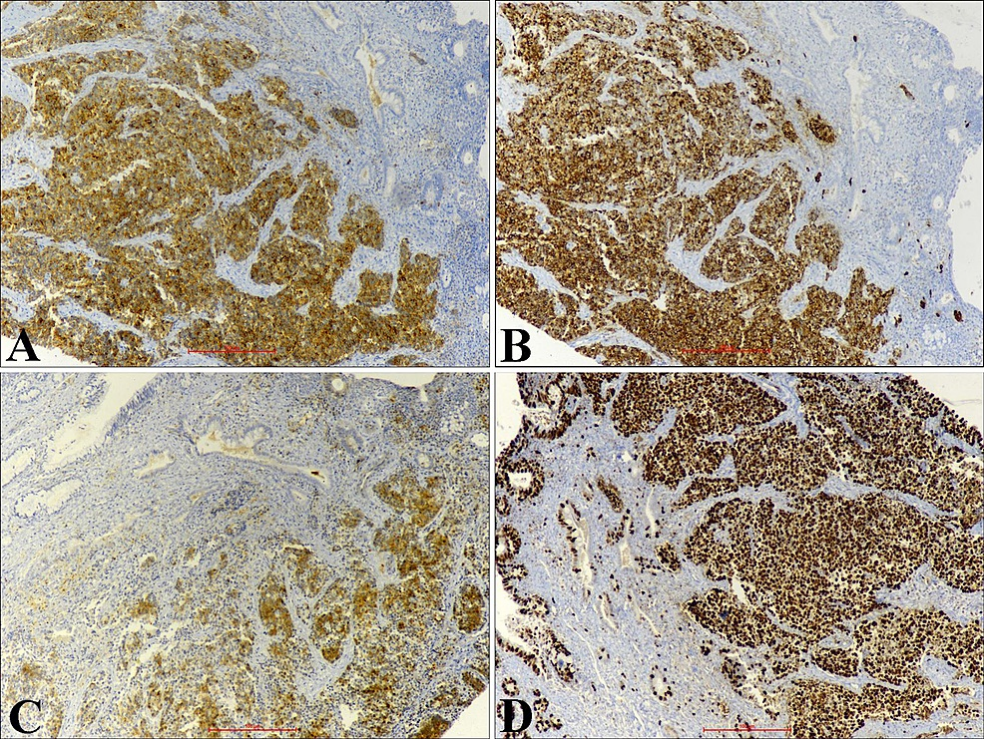
Immunohistochemistry (A) Tumor cells are immunopositive for synaptophysin (x100). (B) Tumor cells are immunopositive for chromogranin (x100). (C) Tumor cells are immunopositive for non-specific enolase (x40). (D) Tumor area shows high Ki 67 proliferating index (x40).

The tumor cells are immunopositive for synaptophysin, chromogranin, and non-specific enolase (NSE). The Ki-67 labeling index was more than 90% (Figures 4A-4D).

We rendered the final histopathological diagnosis of high-grade NE carcinoma, small cell type, cervix. Based on the clinico-radiological assessment and tumor board decision, three cycles of neoadjuvant chemotherapy (carboplatin + etoposide) were given, followed by chemoradiotherapy. After completion of chemoradiotherapy, the patient was lost to follow up.

## Discussion

NENs of the cervix were first described by Albores-Saavedra et al. in 1972 [6]. The exact origin of the NENs of the cervix is unknown [7]. However, argyrophilic cells in ectocervix and endocervix epithelium are considered a potential precursor for NENs [7]. Among NENs of the cervix, SCNEC is the most common (80%) variant, and it constitutes <1% of all female genital tract malignancies [8,9]. The mean age of SCNEC diagnosis is 48.1 years [9]. NECs are more prone to early lymphatic and hematogenous spread and increased risk of nodal metastases even when the tumor is clinically limited to the cervix [8].

Diagnostic accuracy in detecting SCNECs by Pap smear is very low [10]. However, Pap smear shows singly and loosely cohesive groups of relatively uniform small cells [11]. These cells have hyperchromatic nuclei with granular or stippled chromatin, inconspicuous nucleoli, and scant cyanophilic cytoplasm [11]. Nuclear molding, crush artifacts, necrosis, and mitotic figures are also identified [11]. In our case, the patient was middle-aged, and her cervical Pap smear did not reveal any definitive features of NENs.

Histomorphology includes various differential diagnoses such as poorly differentiated squamous carcinoma with small cells, poorly differentiated adenocarcinoma, low-grade endometrial stromal sarcoma, lymphoma, rhabdomyosarcoma, melanoma, myeloid sarcoma, and primitive neuroectodermal tumor (PNET) [11]. McCluggage et al. found that synaptophysin and CD56 are the most sensitive, while chromogranin is the most specific NE immunohistochemical marker [12]. Based on histomorphology and IHC, we exclude all the differential diagnoses in our index case.

NECs are spread by lymphatic and hematogenous routes even if the tumor is clinically confined to the cervix [8]. The tumor was localized to the cervix in the present case, with multiple lymph node metastases. The 2018 FIGO staging system incorporates imaging such as computed tomography (CT) or PET/CT scan and pathologic findings for tumor staging [13,14].

Castle et al. found that 85% of SCNEC and 88% of LCNEC of the cervix were human papillomavirus (HPV) positive. They also conclude that these tumors may be prevented by the use of prophylactic HPV vaccines [15].

Treatment of NETs of the cervix is a multimodality approach [8]. For early-stage disease, radical hysterectomy is followed by adjuvant concurrent chemoradiation and chemotherapy. The use of the immune checkpoint inhibitor (Nivolumab) with or without stereotactic body radiation therapy (SBRT) is helpful in the management of NECs of the cervix [16,17]. Lyons et al. claim successful treatment of recurrent SCNEC of the cervix by the use of trametinib, a MEK inhibitor [18].

Locally advanced disease by concurrent chemoradiation and chemotherapy and palliative chemotherapy for the metastatic tumor is recommended [8]. Since our case was clinically FIGO stage IIA, three cycles of concurrent chemoradiation followed by chemotherapy were administered. The tumor stage is the most critical poor prognostic factor and other independent prognostic factors such as age, tumor size, lymph node metastases, and pure small cell histology [8].

In the index case, poor prognostic factors were tumor stage, lymph node metastasis, and pure small cell histology. Overall, the prognosis remains poor despite multimodal treatment strategy, with a five-year survival rate of 36% and a median overall survival between 22 and 25 months [8].

## Conclusions

SCNECs of the cervix are rare with an aggressive clinical course. Since cervical Pap smear is routing screening processing worldwide, pseudo-rosette-like differentiation and nuclear molding are the important cytological findings that may be considered for suspicion of NE tumors by cytopathologists as observed in our case. It is of utmost importance to accurately diagnose and differentiate from the other high-grade cervical carcinoma for better clinical management.
